# A PRELIMINARY EXAMINATION OF SOCIAL MEDIA USE AMONG HISPANIC/LATINX DEMENTIA CAREGIVERS

**DOI:** 10.1093/geroni/igae098.3445

**Published:** 2024-12-31

**Authors:** Sofia Mildrum Chana, Loreli Alvarez, Andres Azuero, Michael Crowe, Frank Puga

**Affiliations:** University of Alabama at Birmingham, Birmingham, Alabama, United States; University of Alabama at Birmingham, Birmingham, Alabama, United States; University of Alabama at Birmingham, Birmingham, Alabama, United States; University of Alabama at Birmingham, Birmingham, Alabama, United States; University of Alabama at Birmingham, Birmingham, Alabama, United States

## Abstract

Hispanic/Latinx (H&L) family caregivers of people living with dementia (PLWD) experience elevated stress levels that can negatively affect their mental health. Caregivers are also prone to loneliness and social isolation and may rely on technology and social media to enhance social ties, seek support or cope with stressors. However, no studies to date have examined social media use patterns in this population. Thus, this analysis presents a preliminary description of social media use habits among H&L caregivers of PLWD (n=45) employing data from the ongoing national-level, NIA-funded study entitled, “Nuestros Días (Our Days).” We examined bivariate associations between social media use, distress from behavioral symptoms of dementia, perceived general stress, and depressive symptoms. Participants (93.3% female, Mage=55.69, SDage=8.75) reported using social media an average of 158.18 minutes (SD=131.44) per day, with Facebook (31.55%) and Instagram (27.8%) being the most used platforms. Average time spent using social media was positively associated with behavioral symptom distress (r=.37, p=.04) and depressive symptoms (r=.41, p=.02). Passive social media use (i.e., consumption of social media content without active engagement, such as ‘doomscrolling’) was positively correlated with behavioral symptom distress (r=.49, p<.001) as well as perceived stress (r=.33, p=.03) and depressive symptoms (r=.36, p=.02). But active social media use (i.e., engagement with content and deliberate interaction with other users) was not significantly correlated with any mental health outcomes. Findings highlight the different contributions of active versus passive social media use to mental health outcomes, with passive use being more closely linked to worsened mental health.

